# Energy Autonomous Sweat‐Based Wearable Systems

**DOI:** 10.1002/adma.202100899

**Published:** 2021-07-11

**Authors:** Libu Manjakkal, Lu Yin, Arokia Nathan, Joseph Wang, Ravinder Dahiya

**Affiliations:** ^1^ Bendable Electronics and Sensing Technologies (BEST) Group James Watt School of Engineering University of Glasgow Glasgow G12 8QQ UK; ^2^ Department of Nanoengineering Centre of Wearable Sensors University of California San Diego CA 92093 USA; ^3^ Darwin College University of Cambridge Silver Street Cambridge CB3 9EU UK

**Keywords:** biofuel cells, energy autonomy, supercapacitors, sweat‐based energy systems, wearable electronics

## Abstract

The continuous operation of wearable electronics demands reliable sources of energy, currently met through Li‐ion batteries and various energy harvesters. These solutions are being used out of necessity despite potential safety issues and unsustainable environmental impact. Safe and sustainable energy sources can boost the use of wearables systems in diverse applications such as health monitoring, prosthetics, and sports. In this regard, sweat‐ and sweat‐equivalent‐based studies have attracted tremendous attention through the demonstration of energy‐generating biofuel cells, promising power densities as high as 3.5 mW cm^−2^, storage using sweat‐electrolyte‐based supercapacitors with energy and power densities of 1.36 Wh kg^−1^ and 329.70 W kg^−1^, respectively, and sweat‐activated batteries with an impressive energy density of 67 Ah kg^−1^. A combination of these energy generating, and storage devices can lead to fully energy‐autonomous wearables capable of providing sustainable power in the µW to mW range, which is sufficient to operate both sensing and communication devices. Here, a comprehensive review covering these advances, addressing future challenges and potential solutions related to fully energy‐autonomous wearables is presented, with emphasis on sweat‐based energy storage and energy generation elements along with sweat‐based sensors as applications.

## Graphical abstract

Sweat‐based energy harvesting and storage devices, along with the key components of sweat‐based energy autonomous wearables systems are comprehensively reviewed. Various considerations such as the power management and the sweat extraction, etc., are discussed for continuous operation of sweat‐based systems.

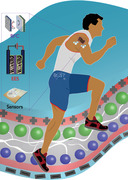

## 1 Introduction

**Figure 1:**
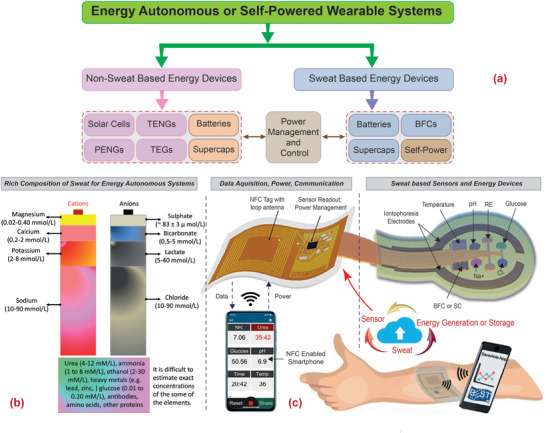
a) Block diagram showing the two routes for achieving energy‐autonomous/self‐powered wearable systems for sweat monitoring along with key devices. b) The major anions and cations present in sweat. c) The general scheme of a sweat‐based health monitoring system showing the flexible patch with multiple sensor to analyze various analytes in sweat and sweat‐based energy generating (BFC) and storage (SC) devices, the smart tag with electronic for data acquisition, power management, control circuitry for automated sweat extraction and finally the wireless powering (via NFC) and wireless data transmission to portable gadget, such as smartphone. The data in (b) are from ref. ^[^ ^83^ ^]^. Image of NFC enabled smart phone and cloud in (c): Adapted with permission.^[^ ^40^ ^]^ Copyright 2020, The Authors, published by The American Association for the Advancement of Science.

Wearable systems incorporating physical, chemical, and biological sensors and actuators have rapidly become an inseparable part of our lives for their use in a wide range of applications, such as personalized health monitoring, wellness‐tracking, early‐warning for COVID‐19, exoskeletons, prosthetics, and interactive systems for augmented/virtual reality.^[^ ^1^, ^2^, ^3^, ^4^, ^5^, ^6^, ^7^, ^8^, ^9^ ^]^ The continuous operation of these systems is juxtaposed with the reliable and sustainable energy sources, currently met through: a) energy harvesters based on mechanisms such as photovoltaics,^[^ ^10^, ^11^, ^12^, ^13^ ^]^ piezoelectricity,^[^ ^14^, ^15^, ^16^ ^]^ triboelectricity,^[^ ^14^, ^17^, ^18^, ^19^ ^]^ and theremoelectricity,^[^ ^20^, ^21^, ^22^ ^]^ etc.; b) energy storage devices such as Li‐ion batteries (LiB)^[^ ^23^, ^24^, ^25^, ^26^, ^27^ ^]^ and supercapacitors (SCs),^[^ ^28^, ^29^, ^30^, ^31^, ^32^, ^33^, ^34^, ^35^ ^]^ etc.; and c) low‐power or near off‐state electronics and algorithms that extend the battery life,^[^ ^36^, ^37^ ^]^ etc. (**Figure** **1**a). Adapting these technologies, a variety of wearable physical, chemical, bio, and optical sensor,^[^ ^3^, ^33^, ^38^, ^39^, ^40^, ^41^, ^42^, ^43^ ^]^ reported in recent years as self‐powered or energy‐autonomous,^[^ ^15^, ^33^, ^44^, ^45^, ^46^, ^47^ ^]^ can rely on energy generators,^[^ ^21^, ^33^, ^48^, ^49^, ^50^, ^51^ ^]^ electrochemical energy storage (EESs) devices,^[^ ^3^, ^26^, ^31^, ^32^, ^33^, ^34^, ^35^, ^44^, ^52^, ^53^, ^55^, ^56^, ^57^ ^]^ wireless power technologies,^[^ ^58^, ^59^, ^60^ ^]^ self‐powered sensors,^[^ ^15^, ^33^, ^44^, ^45^, ^46^, ^47^ ^]^ and hybrid energy system combining both energy generator and EES.^[^ ^61^ ^]^ Several review articles have covered these technologies in detail,^[^ ^47^, ^62^, ^63^, ^64^, ^65^, ^66^, ^67^, ^68^ ^]^ in respective topics such as self‐powered biosensors,^[^ ^69^ ^]^ self‐powered medical sensors,^[^ ^70^ ^]^ enzyme‐based body‐worn devices,^[^ ^71^ ^]^ and other technologies for the environment.^[^ ^72^ ^]^ However, few focused on the biocompatibility, safety, and potential environmental impact such energy‐autonomous systems, which has been a topic evoking increasing interests.

The surge in the use of wearable technologies and the simultaneous global drive toward zero waste, sustainable information and communications technologies, and electrical waste recycling, require that future energy needs be met with sustainable materials.^[^ ^73^, ^74^, ^75^ ^]^ In the case of wearables, there are additional requirements of biocompatibility, and novel form factors that allow wearability (e.g., stretchability, flexibility, washability). Many of the current energy devices use toxic materials and electrolytes, which require attention as the safety of individuals wearing these devices is paramount. For example, the presence of heavy metals including Cobalt and Nickel and the flammable electrolytes (LiBF_4_, LiPF_6_, LiClO_4_) causes toxicity and pollution. Further, heat is produced during the reactions of organic solution and the electrode surface, which is harmful for wearables.^[^ ^76^ ^]^ In this regard, the new approaches that use sweat, by virtue of its rich composition (Figure 1b) for energy generation,^[^ ^40^, ^77^ ^]^ energy storage,^[^ ^35^, ^78^, ^79^ ^]^ and sensing^[^ ^80^, ^81^, ^82^ ^]^ (Figure 1c) are considered very attractive as their integration could lead to fully energy‐autonomous wearable systems. This review article presents a detailed analysis of sweat‐based devices for energy generation, storage, and associated powering, leading to safe and sustainable energy‐autonomous wearable systems. It may be noted that sweat is also an excellent biofluid that can provide early signs of chronic diseases (e.g., cystic fibrosis, diabetes, ischemia, gout, and various skin injuries).^[^ ^83^, ^84^, ^85^, ^86^, ^87^, ^88^, ^89^, ^90^ ^]^ In fact, many sweat‐based sensors have been reported in and several review articles have been published in recent years.^[^ ^82^, ^91^, ^92^, ^93^, ^94^, ^95^, ^96^, ^97^ ^]^ Likewise, various energy generators and storage devices developed for powering wearable sensors are discussed in many review articles.^[^ ^33^, ^98^ ^]^ By focussing on sweat‐based energy devices and systems, this comprehensive review complements the previous reviews, supporting discussions on sensing versus energy usage. Fully sweat‐based wearables will be an important step toward safe and sustainable autonomous systems for continuous monitoring of vital health‐related parameters.^[^ ^2^, ^17^, ^18^, ^23^, ^26^, ^45^, ^46^, ^54^, ^55^, ^56^ ^]^

**Figure 2:**
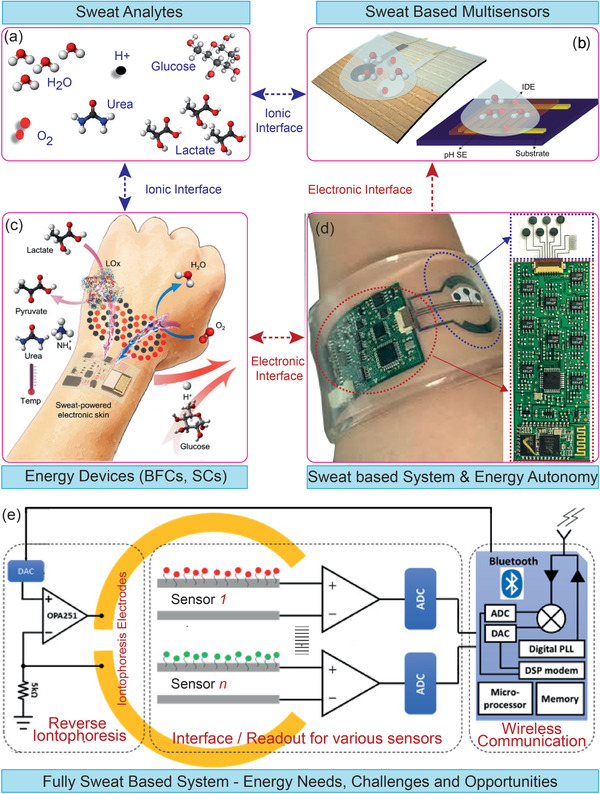
The key phases related to the development of fully sweat‐based energy autonomous system: a) Key sweat analytes, b) sensor that are used for the monitoring of various analytes. c) The energy devices based on sweat. d) The sweat‐based system implementation and the steps toward energy autonomy. The magnified image shows the sensory system. e) Design for realization of fully sweat‐based energy autonomous systems. The structure of this paper follows these key phases related to fully sweat‐based energy autonomous systems. a) Adapted with permission.^[^ ^40^ ^]^ Copyright 2020, The American Association for the Advancement of Science. d) Adapted with permission.^[^ ^90^ ^]^ Copyright 2017, National Academy of Sciences. Magnified image in (d): Reproduced with permission.^[^ ^81^ ^]^ Copyright 2016, Springer Nature.

This review is organized as follows: first, the key components of sweat‐based energy‐autonomous wearables are discussed in Section 2, in which the importance of system‐level integration in wearables is presented. The discussion here also applies to other energy‐autonomous systems where a sustainable means to generate and store energy is very much needed. The flow of the discussion after this section is denoted in **Figure** **2**. Laying foundations, and to provide the reader with the big picture, we briefly introduce in Section 3 the various constituents of sweat, their sensing, and their suitability for use in sweat‐based energy system. Section 4 presents a literature survey of various sweat‐based energy generators, in which we discuss the biochemical mechanisms underlying biofuel cells (BFCs). This is followed by sweat‐based energy storage elements in Section 5, where we review the working principles, including that of SC and batteries, and present some examples. Section 6 distinguishes the traditional energy system with the promising sweat‐based counterpart, along with examples of BFCs and SCs that are used to power sensors and other applications. Future challenges toward the realization of fully energy‐autonomous sweat‐based systems are discussed in Section 7 along with potential solutions. Finally, the key outcomes and future perspectives are discussed in Section 8, whereby we emphasize the need for system power management, which is vital for continuous operation.

Throughout this review, the challenges and potential solutions related to energy‐autonomous wearables are discussed. As an example, a careful assessment of new materials and design considerations for the enhanced performance of sweat‐based energy elements has been presented. We envision that this review article will provide a complete picture of energy autonomous wearable system and other potential applications where sweat equivalent solutions can be used.

## 2 Key Components of Energy‐Autonomous Wearable Systems

The three key components of energy‐autonomous wearable systems (Figure 1a) are: a) energy generators or harvesters; b) energy storage devices, and c) system level integration strategies for power management, low‐power or near off‐state ultralow power electronics for data acquisition and control for online sweat monitoring (see Figure 2). These are briefly described below.

### 2.1 Energy Harvesting

The renewable energy harvesting methods explored to power various devices on the wearables include: solar cells,^[^ ^10^, ^11^, ^12^ ^]^ piezoelectric nanogenerators,^[^ ^14^, ^15^ ^]^ triboelectric nanogenerators (TENG),^[^ ^14^, ^17^ ^]^ and thermoelectric generators,^[^ ^20^ ^]^ etc. The ambient or the body's kinetic energy harvested with these methods is used for the powering of sensors and associated electronics. In some cases, these harvesters themselves also act as the sensors and are termed as “self‐powered.”^[^ ^15^, ^43^, ^69^, ^99^ ^]^ In context with sweat, the predominant study of sweat‐based energy generators relies on the operation of BFC,^[^ ^77^ ^]^ a fuel cell that uses enzymes or microbial as catalysts to generate the power from bioelectrocatalytic reaction of common chemicals and metabolites (lactate, glucose, or alcohol) in sweat. These are discussed in detail in Section 3.

### 2.2 Energy Storage

Most commercially available wearables use batteries (e.g., Li‐ion) as a source for continuous power. Often these energy storage devices are bulky, heavy, rigid, poses safety concerns, and hence are undesirable for wearable applications. As a result, flexible, printable, and stretchable batteries (e.g., silver–zinc batteries^[^ ^100^, ^101^, ^102^ ^]^) and fiber or textile‐based devices^[^ ^35^, ^103^ ^]^ are being explored. The current collectors of wearable rechargeable batteries require special attention, as they dictate the mechanical and electrical properties of the cell. To this end, the textile‐based current collectors have been explored. For example, a fully functional wearable textile battery using Ni‐coated polyester yarn as a current collector has been reported.^[^ ^104^ ^]^ These approaches could also involve the coating of pre‐existing textiles with various carbon or redox‐active electrode materials. Most of these energy storage devices use materials that are unsafe for wearables. For example, a vast majority of wearable EES devices use toxic electrolytes (acid, alkali, ionic liquids) and traditional materials. In this regard, the sweat‐based energy storage approach is attractive. For example, the sweat could be utilized as the electrolyte for textile‐based SCs^[^ ^35^, ^78^ ^]^ and stimulated battery electrodes.^[^ ^35^ ^]^ These devices could store the energy generated by BFCs^[^ ^35^, ^105^ ^]^ or other renewable sources, such as solar cells,^[^ ^35^ ^]^ and continuously power the wearable sensors^[^ ^32^ ^]^ or “self‐powered” wearables,^[^ ^47^ ^]^ as discussed later in Section 6. Energy devices based on sweat or other equivalent safe electrolytes could also help tackle the issue of sustainability and environment friendliness.

### 2.3 System‐Level Integration

The local computing and communication in wearables can quickly drain the energy storage devices they use. As a result, low‐power or zero power energy devices for sensing and power management and wireless powering of devices have received considerable attention. Low‐power electronics can dramatically reduce the energy consumption.^[^ ^36^, ^37^, ^106^ ^]^ For example, by steepening the subthreshold slope and subthreshold operation it is possible to reduce the operating voltage and current and hence the power (as power is the product of voltage and current).^[36]^ The low‐power electronics could be further complemented by algorithmic approaches, such as adaptive current limit pulse frequency modulation, which do the job with a fraction of real‐time dataset.^[^ ^107^ ^]^ Likewise, instead of the continuous reading of sensors data, the techniques, such as compressive sensing, change point‐based activity monitoring (CPAM), machine learning, etc., could be used to recognize the critical natural changes (e.g., activity transitions) and adapt the data acquisition and sampling rates to these changes.^[^ ^108^ ^]^ As an example, the employment of CPAM could result in a saving of 74.64% of energy and thus extend the battery life for activity‐aware applications. A detailed discussion about such methods, which are also used in context with electrical vehicles, can be found elsewhere.^[^ ^109^, ^110^, ^111^, ^112^, ^113^ ^]^ The energy requirement for sweat‐based system could vary with the type of electrodes as briefly discussed in Section 3.

## 3 Sweat Analytes and Monitoring

Sweat is an excellent biofluid which has recently been explored for detection of chronic diseases including diabetics using the various analytes presence in the sweat. In this section, we present a short discussion about various sweat analytes and the enzymatic/nonenzymatic sensors reported for their monitoring.

### 3.1 Sweat Analytes

Sweat contains a plethora of chemicals that reflect important information about our physiological activities (Figures 1b and 2). Composed of over 99% of water as the solvent, the constituents of sweat solute can be classified into 3 categories: a) the electrolyte, b) the metabolites, and c) foreign chemicals. The sweat electrolytes contain the basic salts (e.g., potassium (K^+^), sodium (Na^+^), and chloride (Cl^−^)) that make up the majority of the ions in the sweat. Other cations, such as calcium, magnesium, manganese, iron, and copper, and anions such as iodide, fluoride, and bromide, etc., found in sweat have lower concentrations. The sweat pH varies from 4 to 7, reflecting dynamic changes in the sweat composition.^[^ ^114^ ^]^ Such variations have been exploited to develop a noninvasive route for health monitoring, as briefly discussed in Section 3.2.

**Figure 3:**
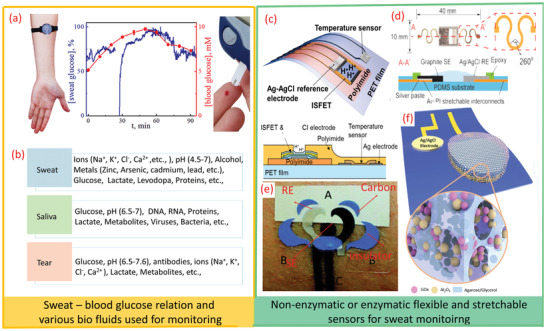
a) Sweat and blood glucose correlation in the same subject. b) Various biomarkers in biofluids, such as sweat, saliva, and tears. c) Scheme of pH and temperature sensors. d) Stretchable pH sensors on PDMS substrate. e) Tattoo‐based ammonium sensor. f) Enzymatic glucose sensor. a) Adapted with permission.^[^ ^120^ ^]^ Copyright 2019, ACS. c) Aadapted with permission.^[^ ^141^ ^]^ Copyright 2017, ACS. d) Reproduced with permission.^[^ ^139^ ^]^ Copyright 2018, Elsevier. e) Reproduced with permission.^[^ ^152^ ^]^ Copyright 2013, Royal Society of Chemistry. f) Reproduced with permission.^[^ ^153^ ^]^ Copyright 2019, Wiley‐VCH.

Sweat metabolites represent another important class of analytes (e.g., lactate, urea, glucose, or uric acid etc.) that could be monitored through noninvasive sweat monitoring methods. The concentration of various analytes in sweat is not the same as in blood^[^ ^115^ ^]^ and several on‐going studies aim to correlate the metabolite concentrations in sweat with their levels in the blood.^[^ ^116^, ^117^, ^118^, ^119^ ^]^ For example, a correlation between sweat and blood glucose levels shows a “lag” of about 8 min.^[^ ^115^ ^]^ Further, based on glucose tolerance, a positive correlation between the rates of glucose concentration increase in blood and sweat has been found (**Figure** **3**a), thus showing the diagnostic value of sweat.^[^ ^120^ ^]^ A remarkable variation in analytes concentrations in sweat and other available body fluids (such as blood, lymph, and cerebrospinal fluid) is obvious with bioanalytes being more abundant in blood compared to that in sweat. The nature of analytes and relative water contents of various body fluids are important determinants for such variations.^[^ ^121^ ^]^ As a result, the correlation between the analytes levels in sweat and other excretory fluids, blood, and lymph need to be established for the effective prediction of health status. Other important sweat analytes, such as the cytokines IL‐6 or the cortisol hormone, offer useful insights into inflammation and stress levels of individuals, respectively.^[^ ^122^ ^]^ Like sweat, other body fluids such as saliva, tear, and interstitial fluid can also be used for the noninvasive monitoring of various biomarkers^[^ ^123^ ^]^ (Figure 3b).

The sensing of foreign chemical compounds in sweat is also of great interest. For example, the ingestion of drinks, foods, and drugs can be reflected by the appearance of related compounds in the sweat. The sweat concentration of alcohol, vitamins, caffeine, antibiotics, heavy metals, or narcotics can vary with the amounts consumed, and thus could be used for personalized nutrition or therapy. In this regard, wearable bioelectronics technology based on electrochemical principles is a prominent research field,^[^ ^35^, ^51^, ^71^, ^124^ ^]^ offering personalized healthcare and fitness management and potential opportunity for telemedicine and remote diagnosis.^[^ ^125^, ^126^, ^127^ ^]^ It is also relevant in the current pandemic situation, as elderly and vulnerable patients could be monitored using wearable wireless sensors.

### 3.2 Sensors for Monitoring Various Sweat Analytes

Monitoring the concentrations of sweat constituents could indicate diseases (e.g., cardiac arrhythmia, hypo/hyperkalemia, hypo/hypernatremia, acidosis, cystic fibrosis, etc.)^[^ ^128^ ^]^ A wide variety of flexible, conformable, and stretchable sensors or sensing arrays have been reported as vehicle for noninvasive detection of the variations in sweat analytes such as glucose, pH, Ca^2+^ ions, Na^+^ ions, sweat rate, levodopa, etc.^[^ ^81^, ^84^, ^92^, ^95^, ^129^, ^130^, ^131^, ^132^, ^133^, ^134^, ^135^, ^136^, ^137^, ^138^, ^139^, ^140^, ^141^, ^142^, ^143^, ^144^, ^145^, ^146^, ^147^ ^]^ As an example, a multisensory patch, containing glucose, lactate, Na^+^, K^+^, and skin temperature monitoring electrodes is shown in Figure 2d. It also shows a compact wireless circuit in a smart wristband design for monitoring the sweat analytes individually without any interference.^[^ ^81^ ^]^

Electrochemical sensors (i.e., potentiometric, amperometric) are especially attractive for skin‐worn wearable applications, owing to their excellent analytical performance, compact instrumentation, and low‐power requirements. Potentiometric sensors are used for monitoring of electrolytes, such as sodium, calcium chloride, and protons, etc. The concentration‐dependent potential response in these wearable sensors is dominated by the Nernst equation and measured by a change in the open circuit potential (OCP) between the working and reference electrodes.^[^ ^132^, ^148^, ^149^, ^150^ ^]^ Here, the flexible solid‐contact ion‐selective electrodes (ISE) replace the traditional bulky ISEs, which contain an inner liquid electrolyte solution that is incompatible with on‐body sensing operations. Further, such classical and rigid liquid‐junction‐based potentiometric sensors do not comply with the softness of the human skin, along with the risk of leakage of the inner solution. The skin temperature and pH value could also affect the performance of wearable sensors due to their changing activity. In this regard, the examples of potentiometric ion‐sensitive field‐effect transistor^[^ ^151^ ^]^ based pH sensor (51.2 mV pH^−1^) with temperature sensors on the same flexible substrate are worthwhile (Figure 3c).^[^ ^141^ ^]^ For conformal contact with the skin, the stretchable and tattoo‐like potentiometric sensors can be very useful. Figure 3d shows one such example of a stretchable pH sensor for real‐time sweat monitoring. Likewise, a tattoo‐based sweat ammonium ion monitoring sensor is shown in Figure 3e.^[^ ^152^ ^]^

### 3.3 Enzymatic Sensors for Monitoring Sweat Analytes

The selective sensing of many metabolites can be achieved by using the corresponding enzymes with amperometric or potentiometric signal transductions. For example, the oxidase enzymes in connection to amperometric enzyme electrodes are useful epidermal applications. In such cases glucose oxidase (GOx) is used for glucose monitoring, lactate oxidase (LOx) for lactate, ascorbic‐acid oxidase for vitamin C, and alcohol oxidase (AOx) to measure alcohol etc. The redox reactions in the biosensing of these metabolites are similar to the ones for BFCs (Section 4) as the latter commonly relies on the use of lactate fuel, owing to significantly higher sweat lactate concentration (compared to glucose or alcohol). In the enzymatic sensors, the degradation or leaching of the enzyme directly into the surrounding biofluid can adversely affect the stability of the sensors. To address this issue, nanoporous enzymatic membranes have been explored as they provide good surface area for molecular or ion diffusion and interactions. The nanoporous membranes also allow sustainable catalytic activities of the sensors while generating reliable performances. Figure 3f shows a schematic illustration of one such porous enzymatic membrane with nanotextured glucose sensors.^[^ ^153^ ^]^ This sensor showed continuous 20 h of operation with minimum drift. For effective immobilization and trapping of the enzyme, this work used an emulsion containing glycerol, agarose, and aluminum oxide (Al_2_O_3_) nanoparticles and created a porous scaffold mixed with the GOx biocatalyst.

Interfacing enzymes with electronic devices is a challenging aspect.^[^ ^154^ ^]^ The fundamental goal here is to achieve efficient electron transfer between the biocatalytic reaction centers and the conductive electrode surface. Based on the enzyme reaction mechanisms, three generations of enzymatic sensing principles have been proposed: the first relies on the reaction of the analyte and the oxygen cofactor with electrochemical detection of the hydrogen‐peroxide reaction product. The second utilizes mediators as artificial electron acceptors (replacing the oxygen) to minimize the oxygen dependence of these devices and to achieve a low‐potential selective detection. The third set uses direct electron transfer between the redox‐active modified enzymes and the electrode surface, hence removing the need for mediators and achieving better selectivity and sensitivity while offering promise for reagent‐less enzyme electrodes. For electrochemical sensing of the sweat metabolite concentration, Chronoamperomery is commonly used with the current response measured 30–60 s after the potential step. Depending on the kinetics of the enzymatic reaction, such response varies linearly with the analyte concentration at low levels and starts to level off at elevated concentrations.^[^ ^155^ ^]^

The success of epidermal biosensors depends on the effective surface immobilization of corresponding enzymes. In particular, the enzyme immobilization has a profound effect on the stability of bioelectronic devices, and the linearity and power density of the biosensors. The enzyme immobilization is commonly achieved with polymers or biopolymers, which entrap the enzyme onto the electrode surface. The reagent layer contains other components, including the mediator, enzyme stabilizer, or crosslinking agent. The nanomaterials can also be incorporated to improve the enzyme wiring.^[^ ^154^ ^]^ Skin worn applications require biocompatible materials that do not leach from the surface. The enzyme immobilization is particularly important in wearable applications involving uncontrolled conditions (e.g., temperature) and fluctuating sweat pH (normally down to pH of 4.5). Without the protective action of the polymeric matrix, the enzyme can be readily deactivated by the varying pH and fluctuating temperatures. Besides entrapping the surface constituents, the coating also excludes electroactive interferences and biofouling macromolecules while imparting mass‐transport limitation to extend the dynamic linear range.^[^ ^156^, ^157^ ^]^

The detection of redox‐active compounds can also be achieved through scanning potential voltammetric techniques, such as cyclic voltammetry (CV), linear scanning voltammetry, square‐wave voltammetry, or normal/differential pulse voltammetry. The electroactive analytes in sweat lead to distinct voltammetric signatures which can be detected directly without the use of a specific receptor.^[^ ^155^ ^]^ For example, the sensitive detection of caffeine, heavy metals, and drugs, such as fentanyl and cocaine in sweat has been achieved using such methods. Electrochemical stripping analysis can be used for detecting trace levels of sweat constituents, particularly heavy metal ions. Such ultrasensitive detection is realized by an initial electrodeposition step to preconcentrate the analyte onto the surface prior to the voltammetric scan.^[^ ^155^, ^158^ ^]^

In the absence of specific enzymes, few sweat analytes such as cortisol or IL‐6 require bioaffinity electrochemical assays for detection. Such assays rely on the use of antibodies, aptamers, or molecularly‐imprinted polymer receptors.^[^ ^159^, ^160^, ^161^ ^]^ The realization of such skin‐based bioaffinity sweat assays is more challenging as they commonly require washing and tagging steps and regeneration of the receptor. Accordingly, these assays are preferred for single‐use impedance detection. The use of redox‐tagged aptamers has recently shown great promise for continuous monitoring applications, as it relies on their reversible folding upon binding to the target analyte.^[^ ^162^ ^]^

## 4 Sweat‐Based Energy Generation

The rich composition of sweat (Figure 1b) makes it attractive for the generation of energy needed to power the sensors, discussed in Section 3 to detect various sweat analytes. In this regard, the enzymatic BFCs^[^ ^163^, ^164^, ^165^, ^166^, ^167^, ^168^, ^169^ ^]^ offer an attractive solution as they could be easily integrated with tattoo‐type sensors and electronics and provide sufficient energy. For example, the power required for wearable or tattoo‐type systems, comprising of sensors, electronic readout and interface, and wireless communication, is typically in the order of mW.^[^ ^170^ ^]^ The energy required by an application‐specific integrated circuit used with wearable sensors can be lower than 200 µW.^[^ ^36^, ^37^, ^171^ ^]^ The wearable BFCs can provide a compact power supply with the theoretical power density of nearly 0.1 mW for 10 × 10^−3^ m lactate.^[^ ^172^ ^]^ Mainly developed with biocompatible materials (for electrodes and electrolytes) they offer significant advantages over body movement or light‐based energy generators.

**Figure 4:**
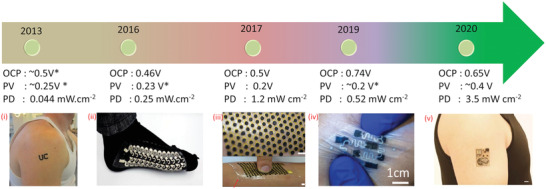
Advances in sweat‐based BFC: i) tattoo BFC. ii) Screen printed BFC on socks. iii) Stretchable BFC in human hand. iv) Stretchable BFC on buckypaper pinched by a human hand. v) PPES on arm. i) Reproduced with permission.^[^ ^182^ ^]^ Copyright 2013, Wiley‐VCH. ii) Reproduced under the terms of the ACS Author Choice with CC‐BY license (https://pubs.acs.org/page/policy/authorchoice_ccby_termsofuse.html).^[^ ^189^ ^]^ Copyright 2016, ACS. iii) Reproduced with permission.^[^ ^77^ ^]^ Copyright 2017, Royal Society of Chemistry. iv) Reproduced with permission.^[^ ^180^ ^]^ Copyright 2019, Wiley‐VCH. v) Adapted with permission.^[^ ^40^ ^]^ Copyright 2020, The Authors, published by AAAS.

BFCs have a rich history of more than five decades and recent advances related to the wearable BFCs, as shown in **Figure** **4**. Wearable BFCs (Figure 2) utilize the redox‐active metabolites in sweat as biofuel to generate electricity based on bioelectrocatalytic reactions.^[^ ^173^ ^]^ Since the catalysis of such chemicals does not require high selectivity to specific compounds, the BFCs can be obtained with a wide range of catalysts and biocatalysts, such as noble metals, enzymes, bacteria, or nanomaterials. Among sweat constituents (Figures 1b, 2, and 3b), the lactate is most attractive for BFCs owing to its relatively high concentration.^[^ ^174^ ^]^ Sweat‐based BFCs commonly rely on an immobilized lactate oxidase (LOx) enzyme on the anode. The enzyme‐electronics interface plays a key role in the exploitation of biocatalytic reactions to develop such wearable energy harvesters.^[^ ^71^ ^]^ The immobilized LOx can effectively oxidize lactate into pyruvate in the presence of electron‐accepting mediators. Ferrocene and quinone derivatives, such as dimethyl ferrocene or 1,4‐naphthoquinone (NQ), or tetrathiafulvalene (TTF), are thus commonly used as mediators to aid the electron transfer, by shuttling electrons between LOx and the electrode, and enhance the power density.^[^ ^175^, ^176^ ^]^ The potential of the anode is thus mostly dominated by the selection of mediators, which dominate the onset potential of the oxidation reaction on the anode. The successful selection of the mediators depends on the specific system environment, where the mediator should be compatible to the electrode surfaces, and can be immobilized without leaching. Furthermore, the mediator, as a highly redox‐reversible compound, should offer a lower reaction potential along with higher biocatalytic currents compared to the oxygen‐based lactate oxidation reaction. The enzyme is confined on the bioanode via physisorption or covalent bonding to ensure high activity and stability, e.g., by cross‐linking of its amine group to carboxylated carbon nanotubes (CNTs).^[^ ^177^, ^178^ ^]^ Polymers or biopolymers, such as Nafionor chitosan, are often added onto the anode to physically protect the enzyme and prevent the leaching.^[^ ^179^ ^]^

The anode oxidation reaction is usually complemented by the oxygen‐reduction reaction (ORR) on the cathode. Platinum (Pt) is the most common inorganic catalyst that facilitates the ORR reaction in the sweat. Some reports also indicate that Pt alloy with other metals could increase catalytic efficiency and stability.^[^ ^180^, ^181^ ^]^ Biocatalysts such as the enzyme Bilirubin oxidase (BOD) are also commonly used for the ORR reaction. BOD can facilitate direct electron transfer to CNTs and hence exhibit high performance comparable to Pt‐based cathodes. The BOD reaction can be improved further by mediators such as 2,2'‐azino‐bis(3‐ethylbenzothiazoline‐6‐sulfonic acid and compounds such as protoporphyrin IX that aid the surface alignment of the enzyme.^[^ ^180^, ^181^ ^]^ Many previous studies have reported the membrane‐less BFCs where the anodic and cathodic catalysts are functionalized onto separate electrodes. The BFCs adapting such cell structure, with the anodes and cathodes configurated in planar interdigitated layout, can be used epidermally to harvest energy from sweat.

**Figure 5:**
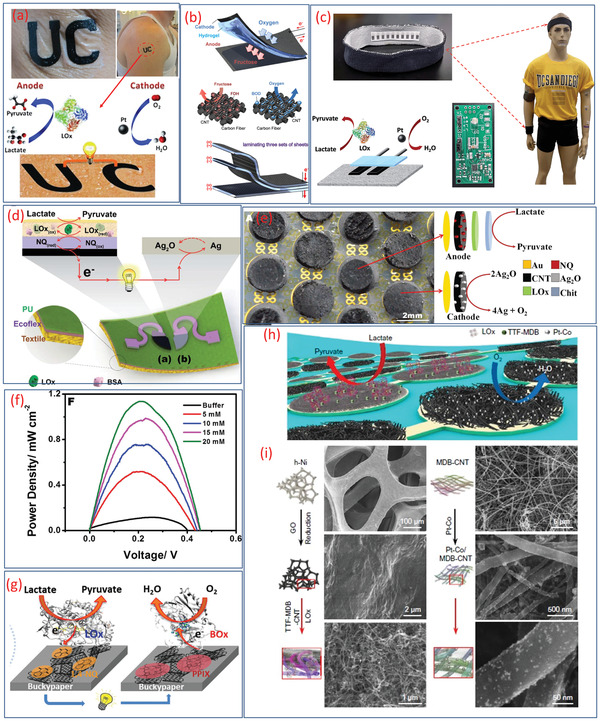
a) Illustration of an epidermal tattoo‐based BFC. b) Schematic representation of carbon‐fiber‐based BFC showing the reactions in the anode and cathode and multilamination of layered BFCs. c) Wearable textile BFCs integrated headband/wristband with a printed circuit board for energy management, and the illustration of redox reaction (lactate oxidation at bioanode and oxygen reduction at the cathode). d) Schematic of the stretchable lactate‐based textile BFC (cross‐section showing the layered stretchable substrates) and the redox reactions that occur on the electrodes. e) Image and schematic illustration of the electrochemical reaction in the anode and cathode of a stretchable BFC. f) Power density curve of the BFC. g) Schematic illustration of the redox reaction including lactate oxidation at the anode and O_2_ reduction at the cathode of BFC on buckypaper. h) Schematic of a soft BFC array consisting of LOx‐modified bioanodes and Pt alloy. i) Preparation steps of nanoparticle modified bioanode assembled with porous h‐Ni and Pt alloy cathodes. a) Reproduced with permission.^[^ ^182^ ^]^ Copyright 2013, Wiley‐VCH. b) Reproduced with permission.^[^ ^185^ ^]^ Copyright 2013, Elsevier. c) Reproduced with permission.^[^ ^186^ ^]^ Copyright 2014, Royal Society of Chemistry. d) Reproduced with permission.^[^ ^189^ ^]^ Copyright 2016, Royal Society of Chemistry. e,f) Reproduced with permission.^[^ ^77^ ^]^ Copyright 2017, Royal Society of Chemistry. g) Adapted with permission.^[^ ^180^ ^]^ Copyright 2019, Wiley‐VCH. h,i) Reproduced with permission.^[^ ^40^ ^]^ Copyright 2020, AAAS.

**Figure** **5**a shows an example of energy generation from human perspiration using a tattoo‐based BFC.^[^ ^182^ ^]^ The developed noninvasive epidermal temporary transfer BFC (tBFCs) uses a TTF‐CNT composite on top of carbon‐based anode electrode. Further, a biocatalytic LOx/albumin layer is coated on the top of TTF/CNT to form bioanode with chitosan as the capping layer. The TTF/CNT composite enables an effective electron shuttle and the TTF exhibited a low oxidation potential and a stable performance for electron transfer mediation. The LOx functions as a selective oxidation agent of lactate present in the perspiration. Chitosan crosslinked with glutaraldehyde prevents the leaching of enzymes and mediators from the electrode surface onto the underlying skin. A printed carbon electrode, modified with Pt black and coated with a protective biocompatible Nafion layer, has been used in this work to develop the cathode. The Nafion layer avoids direct platinum‐skin contact. The modified LOx anode (mediated with TTF) showed the onset oxidation potential of −0.1 V and the peak potential of +0.14 V (vs Ag/AgCl). Upon coupling with the Pt black cathode, the BFC demonstrated an OCP of ≈0.5 V with peak power at the potential of ≈0.25 V. The tBFC exhibited power densities ranging from 5 to 70 µW cm^−2^. The observed power may be useful for operating chemiresistive sensors fabricated using conductive materials or chemiresistive‐based sensors due to their low power requirement.^[^ ^32^, ^139^, ^183^ ^]^ It is worth noting that the power of the tBFC varies based on the lactate sweat concentration, reflecting the fitness level of the test subjects. The subjects with lower fitness levels are less adaptive in changing the oxygen supply to the body, hence exhibit more anaerobic metabolism that produces higher lactate concentration during exercise.^[^ ^182^ ^]^ The direct correlation of generated power with physiological activity also allows the tBFC to be used as a self‐powered sensor—with variation in power providing a measure for the lactate level.^[^ ^182^ ^]^

The power density of these devices could be improved to match the energy requirements of sensors and electronics by using new electrode materials and innovative fabrication methods. In this regard, the fabrics and textile‐based sensors are attractive as they are user friendly and compact.^[^ ^14^, ^184^ ^]^ Layered flexible BFC has also been reported in a biobattery configuration, which uses fabric‐based bioanode for fructose oxidation, hydrogel sheets containing the fructose fuel as the electrolyte, and fabric‐based oxygen diffusion biocathode (Figure 5b).^[^ ^185^ ^]^ High energy density and stretchability are the key challenges for this type of biofuels. One of the approaches to address this challenge is to use a stack of BFC layers (Figure 5b), which produces 2.09 V (as compared to 0.74 V of a single cell) and a similar current as for single BFC.^[^ ^185^ ^]^ The maximum power produced by these stacked BFCs is 0.64 mW (2.55 mW cm^−2^) at 1.21 V.^[^ ^185^ ^]^

For high power density, the textile‐based BFC (100 µW cm^−2^ at 0.34 V) has also been developed, as shown in Figure 5c.^[^ ^186^ ^]^ It relies on the oxidation reaction of sweat lactate at the bioanode and oxygen reduction at the cathode for generating electricity. During in vitro experiments, the power density of cloth BFCs was observed to be 100 µW cm^−2^ at 0.34 V. This research opens new directions to the development of novel BFC in textiles form‐factor and has huge potential in textile‐based wearable sweat monitoring systems.^[^ ^140^, ^187^, ^188^ ^]^ However, the inherent low voltage poses difficulties in the direct powering of electronics. As the epidermal BFCs are powered by perspiration from the skin, a serial connection between BFC units is unpractical because the sweat‐wetted skin can short‐circuit the electrodes. To this end, low‐power, high‐efficiency DC–DC voltage booster can be employed to power the electronics that require >3 V, as demonstrated by direct powering of a light‐emitting diode (LED) or a digital watch used for continuous glucose monitoring.^[^ ^162^ ^]^ Such display‐based sensors have significant applications in personal healthcare, fitness management, and clinical research. Another highly stretchable textile‐based BFC, developed by screen printing of stretchable inks with CNT and Ag_2_O/Ag electrodes, is illustrated in Figure 5d. This BFC offers stable power output after 100 cycles of 100% stretching and produces power densities of 160 and 250 mW cm^−2^ with single‐enzyme and membrane‐free configurations respectively.^[^ ^189^ ^]^ In addition to powering the sweat monitoring sensors, such BFC can be integrated in socks for potential application in wound pressure monitoring or to power biodegradable piezoelectric pressure sensors.^[^ ^106^, ^190^ ^]^

In addition to the challenges related to improving the power density, the mismatch between the mechanical properties (e.g., underlying electrodes are stretchable, but the overlaying active layers are nonstretchable) can lead to gradual delamination and leaching of the layers. Steps toward addressing such challenges include “island‐bridge” BFC configuration with pellet‐based island electrodes (Figure 5e).^[^ ^77^ ^]^ This approach includes high‐surface‐area CNT–LOx pellets with NQ as the mediator for the anode and Nafion‐silver (I) oxide (Ag_2_O) pellets as the cathode. The BFC obtained in this manner shows an OCP of 0.5 V, and a high‐power density of nearly 1.2 mW cm^−2^ at 0.2 V. The major advantages of interdigitated electrode design with serpentine interconnects for the circular anodic and cathodic electrode “islands” are: i) reduced internal resistance (hence decrease the power loss), ii) lower chances of electrodes short‐circuiting, and iii) high stretchability via uniform distribution of the stress.^[^ ^77^, ^191^ ^]^ In addition, the 3D porous structured cathodic and anodic electrode pellets enable high loading of active components. The power density of this BFC increased nearly linearly with increasing lactate concentration (Figure 5f) and the power density is ≈10 times than previously reported BFC.^[^ ^77^ ^]^ The energy harvested by the BFC was used to charging a capacitor (2.2 mF, 3.V at ≈53 s) to operate a Bluetooth Low Energy (BLE) radio device. Another example of a stretchable “island‐bridge” type structure, shown in Figure 5g, is the screen‐printed BFC using BOD‐based ORR cathode.^[^ ^180^ ^]^ The highly conductive and high‐surface‐area Buckypaper used in this work endowed the high double‐layer capacitance to the electrode, which helps in terms of high‐power pulsed discharge. Leveraging the high ORR onset potential of BOD cathode (≈0.6 V vs Ag/AgCl), this BFC harvested OCP of 0.74 V (best, so far in BFC) and a power density of 0.52 mW cm^−2^ in 10 × 10^−3^ m lactate environment.^[^ ^180^ ^]^

The challenges related to improving the power density, poor stability, and short lifetime have also been addressed recently via sweat lactate BFCs. Using TTF‐Meldola blue (MDB)‐mediated LOx anode and cobalt‐doped Pt nanoparticles in the ORR cathode, the developed BFC produced open‐circuit potential of ≈0.6 V and maximum power outputs of ≈2.0 and ≈3.5 mW cm^−2^ in 20 and 40 × 10^−3^ m lactate solutions, respectively for a fully perspiration‐powered electronic skin (PPES).^[^ ^40^ ^]^ The reported power density is the best so far for the sweat‐based BFC (Figure 4). A summary of materials used for sweat‐based biofuel cells and their performnaces are given in **Table** **1**. The redox reaction on the electrodes of this BFC leads to a stable current for electrical loads (the redox reactions of the bioanode and cathode are shown in Figure 5h). The preparation steps of the bioanode and cathode are shown in Figure 5i. This monolithic integration of 0D to 3D helps to obtain the optimal energy performances. In fuel cells, the high electrochemical active surface area (ECSA) of the Pt nanoparticle is one key parameter to enhance the energy performances.^[^ ^192^ ^]^ In this regard, the Pt and Pt‐Co nanoparticle‐coated MDB–CNT electrodes showed higher ECSA of 170 and 210 times compared to the bulk Pt electrodes and it could be one of the reasons for the high power density of this fabricated BFC.^[^ ^40^ ^]^ This BFC‐based PPES was able to power the sensors and wireless data transmission device. In previous work, we used flexible graphite polyurethane SC with a solar cell to power the motors and electronic skin of a robot.^[^ ^34^ ^]^ BFCs could be an attractive alternative for solar cells in such cases.^[^ ^34^, ^40^ ^]^ Moreover, in our previous study, we noted that graphene or ITO‐based electrode may require much lower power ≈20 nW cm^−2^ and 100 µW cm^−2^ respectively.^[^ ^33^, ^193^ ^]^ This means, the touch sensors based on these materials will consume power 3.9 µW for 1.5 m^2^ surface (equivalent adult human body) of a robot^[^ ^3^, ^33^ ^]^ and this much power is within the reach of BFCs (Figure 4). It is worth noting that due to the difference in the characterization methods (e.g., chrono methods vs scanning methods, different scan rates), the power rating of such BFC devices may not be entirely accurate. More standardized characterization for the power of BFCs, similar to that of solar cells, is yet to be proposed and adopted. Indeed, the performance of the BFC varies with individual user due to their different sweat composition and pH values. The composition of sweat as well as the sweat rate are dependent on the physiological status of the user and the location, which have been studied in previous works.^[^ ^194^, ^195^ ^]^ The performance of the biofuel cell thus changes accordingly.^[^ ^182^ ^]^ To mitigate for such uncertainty, the system integration of energy management components with compatible storage units are needed to ensure the reliable operation of the system.^[^ ^196^, ^197^ ^]^ With proper energy management via storing excess power, when lactate concentration is high for later use, and regulating the output of the biofuel cell, the constant delivery of power to the electronics can be maintained.

**Table 1:** The materials used for the fabrication of body‐fluid‐based BFCs and their performances

Electrodes materials		Fuel	OCP [V]	Power density [mW cm^−2^]	Ref.
Bioanode	Cathode				
CNT/TTF/LOx/Chit	CNT/Pt/Nafion	Lactate	≈0.5	0.044	^[^ ^182^ ^]^
CNF/MWCNT/FAD‐GDH/ 9,10‐phenanthrenequinone	CNF/MWCNT/Lacasse	Glucose	≈0.434	0.027	^[^ ^213^ ^]^
CF/CNT/FDH	CF/CNT	Fructose	0.74	0.95	^[^ ^185^ ^]^
Au/CNT/GOx	Au/Lacasse	Glucose	0.23	0.030	^[^ ^166^ ^]^
CNT/ 1,4‐naphthoquinone/GOx+BSA/Chitosan	CNT/Pt/Nafion	Glucose		≈0.012	^[^ ^147^ ^]^
Au/CNT/NQ/LOx/Chit	Au/CNT/Ag2O	Lactate	0.5	1.2	^[^ ^77^ ^]^
Buckypaper/MWCNT/ 1,4‐naphthoquinone (1,4‐NQ)/LOx	Buckypaper/MWCNT/BOx	Lactate	0.74	0.520	^[^ ^225^ ^]^
CNT/ TTF.TCNQ/LOx	Pt/Nafion	Lactate	0.67	0.010	^[^ ^186^ ^]^
h‐Ni/rGO/TTF‐MDB‐CNTs/LOx	Pt alloy‐decorated MDB–CNT	Lactate	0.65	3.5	^[^ ^40^ ^]^
metallic cotton fiber (MCF)/GOx	Metallic cotton fiber (MCF)	Glucose	0.37	1.4	^[^ ^167^ ^]^
Bucky paper/ methylene green/LDH	Bucky paper/BOD	Lactate	0.41	0.008	^[^ ^168^ ^]^
PEDOT/MWCNT/GOx	PEDOT/MWCNT/BOD	Glucose	0.61	0.236	^[^ ^169^ ^]^

## 5 Sweat‐Based Energy Storage

**Figure 6:**
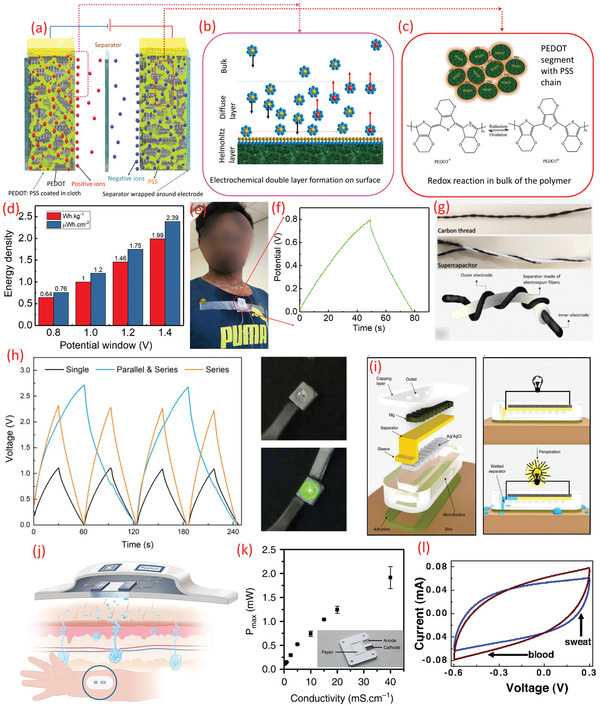
a) Schematic of textile‐based electrodes for SC using sweat as an electrolyte. b) Electrochemical double layer formation on the surface, c) redox reaction on the bulk of the polymer. d) The variation of the energy density of the textile SC (sweat electrolyte) with operating potential window, e) image of the SC on the cloth during exercise. f) The galvanostatic charging–discharging analysis of the textile SC using real sweat. g) Image of twisted configuration wire‐based SC working using sweat equivalent electrolyte. h) Comparison of charge/discharge cycles performances for the combination of SC and its working demonstrated by lighting the LED. i) Exploded view of the sweat‐activated cell with working principle. j) Schematic of the paper battery and its theoretical working. k) The maximum power output density of the paper battery (inset) with the conductivity of the solution. l) CV spectrum of the nanocellulose paper with MWCNT‐based SC in sweat and blood electrolyte. a–f) Adapted under the terms of the CC‐BY Creative Commons Attribution 4.0 International license (https://creativecommons.org/licenses/by/4.0).^[^ ^35^ ^]^ Copyright 2020, The Authors, published by Wiley‐VCH. g,h) Reproduced under the terms of the CC‐BY Creative Commons Attribution 4.0 International license (https://creativecommons.org/licenses/by/4.0).^[^ ^78^ ^]^ Copyright 2020, The Authors, published by Springer Nature. i) Reproduced with permission.^[^ ^79^ ^]^ Copyright 2016, The Authors, published by Springer Nature. j,k) Reproduced under the terms of the CC‐BY Creative Commons Attribution 4.0 International license (https://creativecommons.org/licenses/by/4.0).^[^ ^198^ ^]^ Copyright 2019, The Authors, published by Springer Nature. l) Reproduced with permission.^[^ ^199^ ^]^ Copyright 2007, National Academy of Sciences.

There are many positive and negative ions present in sweat^[^ ^83^ ^]^ (the majority being that of Na^+^ and Cl^−^), which could be used as the electrolyte (acid/basic) for energy storage devices, such as batteries or SC.^[^ ^32^, ^34^ ^]^ Among few examples reported for sweat‐electrolyte‐based electrochemical storage include our previous work, where we noted that these ions could be involved in the electrochemical reaction in a textile‐based electrode of a SC (**Figure** **6** a).^[^ ^35^ ^]^ In the sweat equivalent solution, the SC exhibited a specific capacitance of 8.94 F g^−1^ (10 mF cm^−2^) at 1 mV s^−1^. We observed that the choice of active electrode, i.e., poly(3,4‐ethylenedioxythiophene):poly(styrenesulfonate) (PEDOT:PSS) doped with dimethyl sulfoxide has an appreciable impact on the electrochemical performance due to the coupling between electron and ions transport.^[^ ^35^ ^]^ During charging/discharging, both the electrochemical double‐layer capacitance (EDLC) formation and redox reactions of PEDOT:PSS in conjugated polymer film occurs and contributed to the high capacitance of SCs (Figure 6b,c).^[^ ^35^ ^]^ Further, the energy and power densities of the SC increased with increasing potential window and in sweat equivalent electrolyte the SC operated a maximum potential window of 1.31 V (Figure 6d). At this potential, the specific capacitance of 5.65 F g^−1^ was obtained with the energy and power densities of 1.36 W h kg^−1^ and 329.70 W  kg^−1^, respectively. To demonstrate the use of these SCs in real life, they were attached to the shirt so that they could be operated with human sweat during exercise (Figure 6e). The observed galvanostatic charging/discharging performances of the SC (Figure 6f) show that the amount of sweat influences the performance of SCs, which were developed on the cellulose substrate to allow easy absorbance of even the small amount of sweat on the surface of the active electrode. The investigation about the influence of the volume of sweat showed that the SC's (size −0.5 × 2 cm^2^) exhibit excellent performance at 100 µL. For fully wetted the SC, in real human sweat the energy and power densities were 0.25 Wh kg^−1^, and 30.62 W  kg^−1^ at 0.8 V, respectively. The comparison of the performances of the SC in real and sweat‐equivalent solutions (artificial sweat) results in almost similar behavior. The performances of these SCs could be further enhanced by using transition metal oxides and composited with carbon‐based electrodes.^[^ ^33^ ^]^

Another example is the twisted wire/fiber type SC developed using a carbon‐based thread functionalized with a conductive polymer (polypyrrole) and sweat equivalent electrolyte (Figure 6g).^[^ ^78^ ^]^ This fiber‐based SC exhibit a specific capacitance of 2.3 F g^−1^, an energy and power density of 386.5 mW h kg^−1^ 46.4 kW kg^−1^, respectively. This SC shows an insignificant variation of the specific capacitance under bending angles up to 180°.^[^ ^78^ ^]^ By integrating the fiber‐based SC in series or parallel configuration it is possible to use these fiber‐based SC for applications, such as lighting the LEDs (Figure 6h).^[^ ^78^ ^]^ In these sweat‐based SC, while the can be replenished, the mechanical stability of the used material is an issue that may deteriorate the performance—especially during washing.^[^ ^35^ ^]^ More investigations are needed to obtain highly stable materials and the ones that lead to high energy density for practical applications.

Sweat‐activated cell (SAC) as a battery technology has also been reported recently.^[^ ^79^ ^]^ Embedded in soft, microfluidic structures, they have been explored to monitor sweat analytes (pH, chloride) and heart rate. The schematic representation of SAC, along with various components, is shown in Figure 6i. In a sweat electrolyte‐based SC, such as the ones discussed above, the cellulose/polyester blend is used as the separator so that it is wet during sweating and can act as a carrier of electrolyte containing the ions (Figure 1b). However, the SACs use a dry cellulose membrane impregnated with NaCl to act as the separator. Without sweat, the SAC is in open circuit condition due to the dry separator (Figure 6i) and during sweating the separator absorbs the sweat via a wicking sleeve in direct contact with the skin to close the circuit and produce usable electrical energy (Figure 6i). Thus, sweat acts as both an activating cue and a natural, biocompatible electrolyte. The SAC possesses a specific capacity (≈67 Ah kg^−1^) and can provide sufficient power for about 5 h, comparable to that of a commercial cell (CR2032; specific capacity ≈73 Ah kg^−1^). The high energy density of this cell is due to the electrode materials (Mg as the anode) and the dry nature of the system in its inactivated mode.^[^ ^79^ ^]^

The paper‐battery is another interesting concept, particularly for self‐powered sensors. In this approach, the electrode deposited on a paper is activated when the body fluid acts as an electrolyte. In one such example (Figure 6j),^[^ ^198^ ^]^ the battery uses Mg as anode and AgCl as the cathode as shown in the inset of Figure 6k.^[^ ^198^ ^]^ The sweat patch battery could operate in the conductivity range of 5–160 × 10^−3^ m equiv. NaCl. The power output of the battery and the variation in conductivity of the electrolyte, shown in Figure 6k, indicates that the paper battery could act as sensors or store energy to operate the electronics.^[^ ^198^ ^]^ In addition to sweat, the performance of such energy storage device was also evaluated with blood too. For this, a SC was developed with nanoporous cellulose paper embedded with MWCNT, and the comparison CV spectrum is shown in Figure 6l.^[^ ^199^ ^]^ The specific capacitance of the SC in sweat is 12 F g^−1^, which is lower than the value (18 F g^−1^) with blood.^[^ ^199^ ^]^ A summary of sweat‐based energy‐storage devices is given in **Table** **2**.

**Table 2:** The materials used for the fabrication of body fluid‐based energy storage devices and their performances

Devices	Materials	Sweat‐related electrolytes	Performances	Ref.
SC	Cellulose‐polyester clotPEDOT: PSS	Artificial sweat Real human sweat	ED and PD: 1.36 Wh kg^−1^ and 329.70 W kg^−1^ at specific capacitance is 5.65 F g^−1^. ED and PD: 0.25 Wh kg^−1^, and 30.62 W kg^−1^, respectively, at 0.8 V	^[^ ^35^ ^]^
SC	Carbon thread/polypyrrole	Artificial sweat	Specific capacitance 2.3 F g^−1^. ED and PD: 386.5 mW h kg^−1^ 46.4 kW kg^−1^	^[^ ^78^ ^]^
Sweat activated battery	Mg as anode and Ag/AgCl as Cathode	Sweat	(≈580 Wh kg^−1^ with respect to anode at 1.6 V. Specific capacity: ≈67 Ah kg^−1^)	^[^ ^79^ ^]^
SC	Nanoporous cellulose paper/MWCNT	Sweat	Specific capacitance—12 F g^−1^	^[^ ^199^ ^]^

## 6 Routes for Energy Autonomy

Energy autonomy is the key to the next‐generation portable and wearable systems. As shown in Figure 1a, conventional energy devices (generators and storage), as well as sweat‐based energy solutions (generators and storage) have been used for powering of sensors and related components. In some cases, both conventional or sweat‐based energy devices have been used as sensors. This section presents a brief discussion related to the methods that have been explored to power the sweat‐based sensor, and the approach toward fully sweat‐based system (i.e., sweat sensor, sweat energy generation, and sweat‐based energy storage). Even though compact sweat‐based sensors and energy devices are reported, the challenges related to the availability of sweat, electronics design, and system‐level integration are the major challenges for fully sweat powered systems and these are discussed in the following subsections.

### 6.1 Sweat Sensor Powered with Conventional Energy Devices

**Figure 7:**
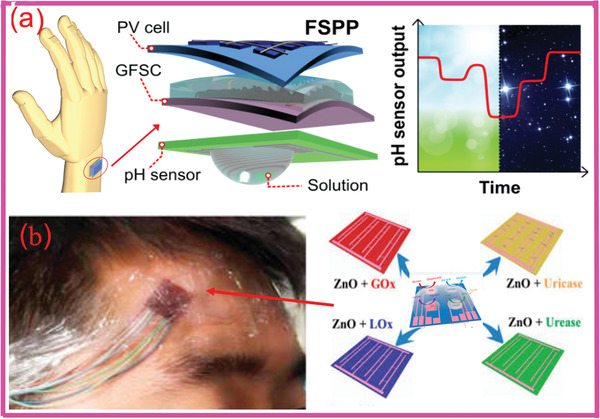
a) Powerpack for self‐powered wearable pH sensing. b) Self‐powered e‐skin for detecting sweat analytes (lactate, glucose, uric acid, and urea). a) Reproduced with permission.^[^ ^32^ ^]^ Copyright 2018, Elsevier. b) Adapted with permission.^[^ ^15^ ^]^ Copyright 2017, ACS.

As briefly mentioned in Section 1, one of the most common strategies currently employed for powering the sweat sensors involves using renewable sources and EES. This includes generating energy from external sources and storing it in SCs or batteries. One such example involves charging batteries using solar cells to power the “smart watch” along with a glucose sensor.^[^ ^162^ ^]^ Another example shown in **Figure** **7**a, is a flexible self‐powered pack with solar cells charging a SC to power a flexible chemiresistive pH sensor. Another recent example, combining the above two approaches, involves using miniaturized solar cell itself as touch sensors—thus leading to simultaneous sensing and energy generation by the same device and to the self‐powered autonomous system that generates enough energy for electronics too.^[^ ^43^ ^]^ Such solutions explored for sweat‐sensors are presented below.

### 6.2 Self‐Powered Sweat Sensors

**Figure 8:**
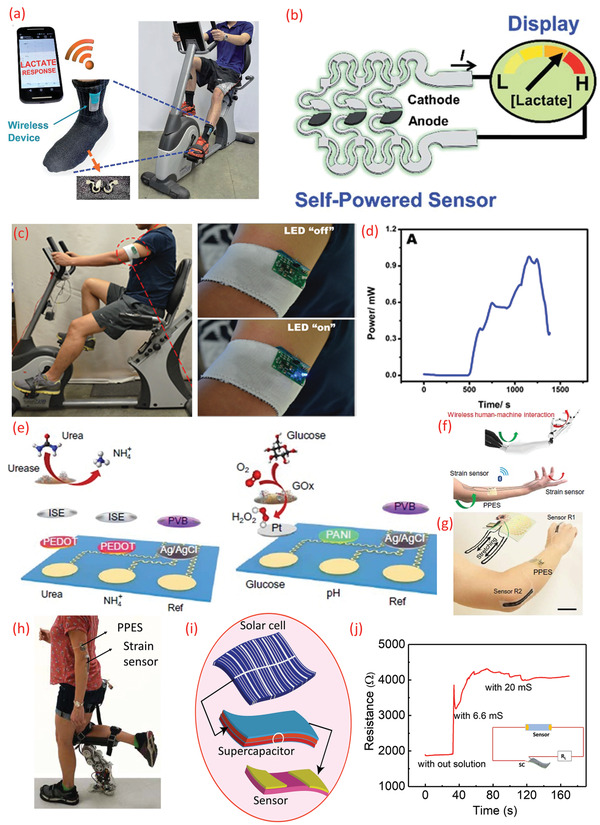
a) BFC on the socks showing the response of lactase for wearable self‐powered sensors during exercise. b) Schematic diagram of the integrated “scavenge‐sense‐display” system for textile BFC. c) Real‐time performances of a stretchable BFC. d) Real‐time scavenging of electrical energy by stretchable BFC in a human subject. e) Schematic of a sensor array (top: urea and NH_4_^+^, bottom: glucose and pH) for simultaneous monitoring of various analytes in sweat. f–h) Schematic representation of perspiration‐powered electronic skin (PPES) for remote human–machine interaction and the PPES integrated with strain sensors. i) Sweat‐based energy storage with hybrid integration of solar cell and chemiresistive sensor for sweat salinity analysis. j) The performance of sweat salinity sensors using sweat electrolyte‐based supercapacitor. a,b) Reproduced with permission.^[^ ^189^ ^]^ Copyright 2016, Royal Society of Chemistry. c,d) Adapted with permission.^[^ ^77^ ^]^ Copyright 2017, Royal Society of Chemistry. e–h) Adapted with permission.^[^ ^40^ ^]^ Copyright 2020, The Authors, published by AAAS. j) Reproduced under the terms of the CC‐BY Creative Commons Attribution 4.0 International license (https://creativecommons.org/licenses/by/4.0).^[^ ^35^ ^]^ Copyright 2020, The Authors, published by Wiley‐VCH.

Due to the autonomous redox reaction, similar to that of BFCs, many enzymatic sensors can be used as self‐powered sensors that do not require applied potential to generate signals. In this case, the energy generator itself acts as the biosensor,^[^ ^15^, ^69^, ^99^ ^]^ as the electrical power output is directly related to the analyte concentration. For example, the BFCs exhibiting the power output proportional to the concentration of the biofuel (i.e., target analyte) could also be used as sensors. Here It is worth highlighting the distinction between such self‐powered sensors and self‐powered autonomous systems, as the former is able to generate the sensing signal autonomously, but still requires an external energy source to power the associated electronics for signal acquisition and data processing.^[^ ^189^ ^]^ Figure 7b shows an example of a self‐powered wearable electronic‐skin using a piezo‐biosensing unit matrix of enzyme/ZnO nanoarrays^[^ ^200^, ^201^, ^202^ ^]^ to detect lactate, glucose, uric acid, and urea, etc. Such self‐powered sensors use the enzyme/ZnO nanowires (NWs) coupling as the key mechanism,^[^ ^15^ ^]^ with output depending on the analyte (urea, glucose/lactate, or uric acid) concentration.^[^ ^15^ ^]^ For self‐powered sensing (e.g., lactate monitoring), the textile BFCs were used as shown in **Figure** **8**a–d.^[^ ^189^ ^]^

### 6.3 Sweat‐Based Energy System for Sweat Sensors

The flexible solar cells, batteries, and SCs for the self‐powered systems could be replaced with BFCs and sweat electrolyte‐based SCs to obtain fully sweat‐based energy‐autonomous wearable systems.^[^ ^35^ ^]^ As shown in Figure 4, the maximum OCP reported for the BFC is 0.74 V^[^ ^180^ ^]^ and the maximum power density has been obtained for the BFC having OCP of 0.65 V.^[^ ^40^ ^]^ The maximum power density obtained for BFC is 3.5 mW cm^−2^. However, many of the electronic components require high operating voltages than those produced by the BFCs. As a result, additional arrangements, such as connecting BFCs in series and using a DC–DC converter circuit could be explored to obtain higher voltages needed for the operation of BLEs in wireless data transmission or the LEDs, as shown in Figure 8c,d.^[^ ^77^ ^]^ This BFC‐based PPES was able to power multisensors (glucose, urea, NH_4_^+^, and pH) and a wireless data transmission module (a user interface realized via BLE) (Figure 8e). The application of this BFC was also demonstrated in prosthesis by powering a strain sensor on an artificial hand (Figure 8f,g) and real human subjects for human–machine interaction^[^ ^40^ ^]^ (Figure 8h). Such high‐power density BFCs could replace the flexible solar cell in Figure 8i to charge the sweat electrolyte‐based SC or could power the salinity sensor (Figure 8j). Likewise, the BFCs could be used with paper battery^[^ ^199^ ^]^ as discussed previously. The sensing and communication devices used nowadays for applications, such as wearables require hundreds of µW power for smooth operation.^[^ ^77^ ^]^ The studies on BFCs, discussed in previous sections, show that they can produce power density up to 3.5 mW cm^−2^ and yet could power the sweat sensors and electronics in pulsed sessions by charging a capacitor that supplies high power in a short burst. Such operation mode can greatly expand the application of lower power harvesters in such high‐power‐demand systems. In this regard, the example of PPES (including BFC, sensing arrays, converters, amplifiers, and BLE etc.) discussed earlier for sweat monitoring sensors is also noteworthy. However, the power requirement could increase rapidly upon increasing the number of sensing and electronic devices.^[^ ^3^, ^34^, ^139^, ^147^, ^203^ ^]^ For example, wearable sensing systems for sweat monitoring require multiple sensing electrodes (Figure 1c) and the energy requirements could vary with materials they use and the type of sensors employed (includes potentiometric, chemiresistive, amperometric, and ion‐sensitive field‐effect‐transistor‐based sensors).^[^ ^204^ ^]^ Nonetheless, as a future perspective, the sweat‐based SCs or paper battery integrated with BFC will open new avenues for energy autonomy of wearable sensors and associated interface electronics for continuous monitoring (Figures 1 and 2). The sweat‐based energy harvesters could also be considered with other energy harvesters to mitigate the issues, such as limited sweat availability in the human body at times affecting the power generation or storage. For example, motion‐activated TENG integrated with sweat‐based BFC could address provide the required energy in the beginning of an exercise session when there is insufficient sweat.^[^ ^205^ ^]^

### 6.4 Meeting the Energy Needs of Sweat‐Based Wearables

**Figure 9:**
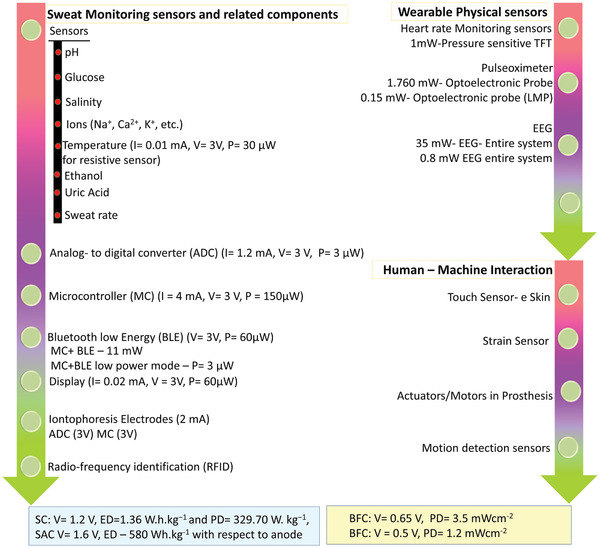
Power requirement for wearable sweat sensors and related components, physical sensors, and examples of sensors in human–machine interaction (prosthetic devices).

**Figure 10:**
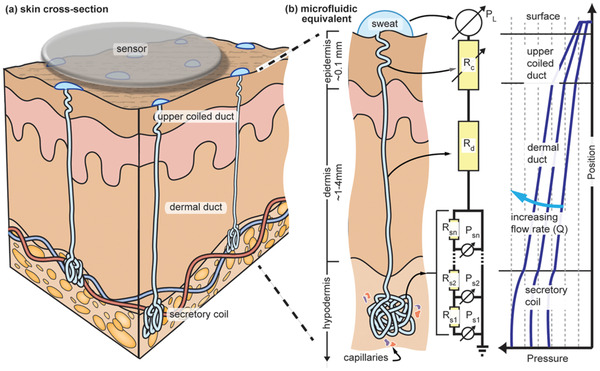
Structure of the human sweat gland showing, a) skin cross‐section, and b) the microfluidic and electrical equivalent of sweat extraction process. *R* and *P* are fluid resistance and pressure differential respectively in the region of interest. a,b) Reproduced with permission.^[^ ^217^ ^]^ Copyright 2015, AIP Publishing.

The sweat sensors and related electronic components in a full sweat‐based platform require power in nW to mW range. For example, the sensors in the system block diagrams of a multisensory patch^[^ ^81^ ^]^ and single sweat conductivity monitoring sensors^[^ ^206^ ^]^ require lower power than the electronic components (e.g., BLE required power in the range of mW). Normally, the operating voltage of a BLE is 3 V and is similar to an ADC (Analog to Digital) or DAC converter. Some of the wearables sensors also use display technology for quick analysis by the wearer. These displays require an operating voltage of 3 V and power in the range of 60 µW. A summary of typical power requirements of sweat monitoring sensors and related components for real‐time applications are summarized in **Figure** **9**. The actual power requirement depends on the specific type of sensors, design, and the material used for the fabrication.^[^ ^68^, ^94^, ^137^, ^139^, ^147^, ^207^, ^208^, ^209^ ^]^ For example, a pH sensor could be either potentiometric, chemiresistive, ion‐sensitive field‐effect transistor, or conductometric/capacitive^[^ ^204^, ^210^ ^]^ and they could be operated with voltage <250 mV and power of the order of nW cm^−2^.^[^ ^32^ ^]^ **Figure** **10** lists a resistive type of temperature sensor that can be operated with 30 µW. The exact operating voltage and current depends on the type of materials such as carbon, conductive polymer, and metal oxides used for the fabrication of sensors.^[^ ^32^, ^95^, ^204^ ^]^ For the development of a fully sweat‐based sensors system, it is important to consider such varied power requirements.

The current BFCs, SCs, SAC can provide a maximum voltage of 0.7 and 1–1.2 V respectively.^[^ ^35^, ^40^, ^77^, ^79^ ^]^ This is sufficient to operate some of the sensors or related devices for a few minutes.^[^ ^40^ ^]^ However, multisensing arrays having displays as in smartwatches, and other components such as iontophoresis electrode, and wireless transmission (Figure 2) may require higher power.^[^ ^33^, ^211^ ^]^ Some of the steps that can help meet the power requirements include using: a) new designs and materials for sweat‐based energy generators and EESs; b) dual‐function devices, e.g., energy devices also acting as sensors; c) suitable power management circuitry allowing sensor operation only when needed; d) ultralower or zero‐power electronics. In the case of EES, the current storage capability can be enhanced by using materials such as metal oxides (CuO, ZnO, IrO_2_, RuO_2_, etc.) instead of polymers or using designs such as asymmetric SCs (hybrid SC),^[^ ^35^, ^212^ ^]^ and e) integrating multiple harvesters that harvest various types of energy simultaneously and synergistically. The operating voltage of sweat‐based SC (maximum 1.2 V) is limited by the presence of water, and enhancing the specific capacitance is an option to improve the performance. In this regard, the metal oxides can help as they show high theoretical specific capacity and could help achieve SCs with higher energy density. Material sustainability is an important factor and in this regard, the use of cellulose‐based molecules and conductive fibers or the development of electrodes from the abundant material (Fe, carbon, Si) can be advantageous.^[^ ^31^, ^213^ ^]^ The mechanical stability (including during washing), charge–discharge cycle life,^[^ ^31^ ^]^ and biocompatibility, etc., are some other factors that require attention during material selection.

The operational life of EES could be enhanced by using advanced switching algorithms, such as the ones used in electric vehicles, i.e., selecting a subset of the battery cells for each current demand and control the discharge current from each cells.^[^ ^109^ ^]^ This discharge is based on the electrochemical properties of the individual cells in the EES, and is carried out in such way that cells were discharged selectively instead of discharged all at once. Different types of EES can be also simultaneously employed to satisfy high‐power demand (via SC) or long‐term storage (via batteries) simultaneously. The operational life of EES can also be improved by exploiting the wireless charging via near field communication (NFC).^[^ ^9^ ^]^ In this case, the sensors should have an integrated antenna so as to harvest energy through electromagnetic fields.

The above solutions to address the issues associated with sweat‐based wearable sensor systems would help advance their use in point‐of‐care and self‐health management as well as open new opportunities. For example, the sweat‐based approach could also help advance the applications, such as prosthesis^[^ ^3^, ^40^ ^]^ where sufficient sweat generated at the socket‐limb interface could be exploited to generate power and hence monitor the health conditions of amputees. The generated energy could also be used for the operation of other sensors on smart prosthetic limbs, such as touch sensors and temperature sensors, etc.^[^ ^3^, ^34^ ^]^ In our previous study, we noted that the graphene‐based transparent touch sensors for e‐skin needed very low power (20 nW cm^−2^) for operation. In this case, the solar cells, placed underneath the transparent touch sensors, were used to obtain the operational power. However, in the case of the prosthesis, these could be replaced or used in combination with BFCs and EES located near the socket of the artificial limb, where sufficient sweat is readily available.

## 7 Challenges and Opportunities

In Sections 3–6, we discussed the sweat‐based energy devices (generator and storage) for sensors and related components. Despite several device level studies, the system‐level integration and powering (not only the sensors but also the electronic components), availability of sufficient sweat, low energy, and power density of energy generator and storage, etc., remain major challenges. While addressing these challenges, one may see new opportunities too. For example, the pursuit for generating sufficient sweat on‐demand could also inspire the modeling of skin with automated sweat extraction processes, which can act as a virtual platform to simulate the performance of sweat‐based wearable devices with controlled variables. Likewise, the sweat‐based energy autonomy has tremendous potential for sustainable and eco‐friendly energy sources needed in application other than wearables. In this section, we discuss these challenges, their potential solutions, and new opportunities for sweat‐based wearable systems.

### 7.1 Variability of Sweat Constituents and Modeling

Sweat analytes‐based prediction of health status faces challenges due to the discrepancies in the physical and biological state of individual subjects, as well as environmental factors during the measurements. Even in healthy subjects, there is interindividual variability in levels of bioanalytes in sweat. Age, sex, physical activity, temperature, sweat gland density, sweating location, diets, socioeconomic state, and ethnic origin are some of the factors responsible for such variations.^[^ ^214^, ^215^ ^]^ The anatomical structures, such as the thicknesses of epidermal, dermal, and hypodermal layers of skin could also affect the availability of sweat due to variations of ductal lengths of the acinar gland.^[^ ^203^ ^]^ These variations should be optimized methodically with suitable modeling prior to the actual device fabrication for sweat analytes‐based prediction of health status or the performance of BFCs and SCs. Modeling is also important from the point of view of the way the measurements are made. For example, sweat‐based wearable sensors generally measure the change in sweat generation rate by skin impedance.^[^ ^216^ ^]^ However, sweat generation rate does not predict actual biomarker sampling intervals without a detailed transport model between sweat glands and sensors. Thus, sweat biosensing will benefit greatly from foundational microfluidic and partitioning models of the eccrine sweat gland and transport mechanism (Figure 10).

Considering that the sensors are expected to be in conformal contact with the skin, the engineering‐driven models (Figure 10)^[^ ^217^ ^]^ could be used to capture the flow rate and biomarker diffusion to determine the effective sampling rate of biomarkers at the skin surface. The models of eccrine sweat gland and transport mechanism between sweat glands and sensors could provide the optimum performance for the sweat‐based platforms. Such models could capture the flow rate and biomarker diffusion to determine the effective sampling rate of biomarkers at the skin surface and can serve as a foundational guide for more advanced models, for further development of fully sweat‐based diagnostics platforms, and for those beginning exploration of new biomarker opportunities in sweat. The understanding of the basic biomarker fluctuation rate is also of great interest as the sensors need to be sampled with sufficient temporal resolution to accurately capture the acute changes of biomarker levels. The undersampling of such fluctuation may result in missing critical information that leads to inaccurate prevention and prediction of symptoms; while the oversampling may result in unnecessarily high energy consumption which negatively impacts the runtime of wearable electronic systems.

The electrical equivalent of the microfluidic models (Figure 10b)^[^ ^217^ ^]^ could be implemented using standard design tools (e.g., PSPICE) that are used for electronics and sensor design. In doing so, one could also see the opportunity to develop a virtual tool, as the electrical models for various sensors and electronic components are generally available and the sweat models could be clubbed with them to simulate the entire platform, i.e., from sweat generation to digital acquisition and inferences. This will also promote the development of user‐centric solutions as the sweat content could vary with individuals’ physiological status or with physical activity. In addition, the models will benefit those pursuing even broader types of on‐skin products such as cosmetics, antiperspirants, garments, wearable electronics, and medical adhesives.

### 7.2 Sweat Extraction

The sweat‐based energy storage, generation and sensors‐based approaches assume that an appropriate amount of sweat will be available all the time, which is not generally the case as environmental factors and physical activity can affect sweat production. For example, there is not enough perspiration in the case of a person with sedentary lifestyle. Availability of minimum amount of sweat is a must for sweat‐based approaches. As a result, methods such as iontophoresis have been explored for sweat stimulation (Figures 1 and 2). Iontophoresis is an electrical process used in clinical practice to deliver drug through the skin,^[^ ^218^, ^219^ ^]^ by applying current between two electrodes with hydrogels containing the sweat‐inducing drugs. The voltage gradient allows the transport of these molecules into the epidermal, where positively charged ions can be repelled from the positive electrode and vice versa, hence achieving the noninvasive transdermal delivery of the drugs.^[^ ^92^, ^220^ ^]^ The iontophoresis electrodes have been successfully used with sweat‐based multisensory patces and could be used to attain higher performance for sweat‐based energy generation and storage devices. The reported sweat‐based systems could be advanced by including automated sweat extraction in the system design and implementation, as shown in Figure 2. This will lead to more energy requirements, but energy available from reported sweat‐based devices discussed in previous sections may be sufficient. Passive (natural) sweating is another route, mostly focusing on the high sweat rate region of the body, such as the fingers.^[^ ^221^, ^222^ ^]^

### 7.3 System Stability and Reliability

The system stability is crucial for sweat‐based energy‐autonomous system. In particular, the mechanical performance of wearable energy devices is of great importance due to requirements, such as flexibility, stretchability, washability, durability, and safety, etc. To simultaneously ensure the mechanical and electrochemical performance of the devices, a mixture of strategies in material engineering and structural innovations must be employed. Currently, the development of wearable, flexible, and stretchable components rely on elementary material innovations (e.g., polymer–particle composites), which sacrifice partially the electrical or electrochemical performance of the devices. Other strategies, involving novel structural engineering (e.g., island–bridge structures) require complex fabrication strategies and larger device footprint to incorporate structures that enable the mechanical resiliency. To eliminate such trade‐offs and develop devices with attractive wearable form factors, further effort is needed in bottom‐up material engineering that develops high‐performance materials with desirable mechanical properties, or in scalable fabrication of carefully optimized structures that fully exploit the limited device footprint, toward advancing the application of sweat‐based devices.

Another aspect of system stability hinges on the biochemical and electrochemical stability of the electrodes and biocatalysts used in sensing, energy generation, and energy storage. Enzymes, as the biocatalyst widely used in the biosensing and energy generation of sweat, deliver different performance in different immobilization environments, enzyme wiring, temperatures, and pH values. As an example, the activity of LOx is sensitive to the pH and temperature, with its activity maximized at 37 °C and pH of 7.4. The stability of the enzyme is highly dependent on its operational conditions and immobilization environment.^[^ ^223^, ^224^ ^]^ Previous study has shown high stability over several weeks of operation upon proper storage.^[^ ^225^ ^]^ Further enzyme engineering can also be employed to develop LOx with higher stability over a wider range of conditions.^[^ ^226^ ^]^ For energy storage purposes, the stability of the electrode in sweat, which is a complex environment with numerous compositions that may cause corrosion or fouling, must be ensured. The retention of water in the system that ensures the longevity of the energy storage devices is also of great importance and is yet to be studied. Recent studies also show that the temperature influences the performances of energy storage devices. In such cases, distinction of influence of heat and excess sweat due to heat on the performances also needs to be studied. In sweat electrolyte‐based SC, it is well studied that volume of sweat has strong influence on the performance of the devices and it also depends on the size of the devices. The amount of sweat produced on a body depends on many factors including gender, age, size, and physiological status.^[^ ^195^, ^227^ ^]^ In one of the studies, it is reported that the insensible water losses for a standardized individual with total of surface area 1.8 m^2^ will be 0.6–2.3 L. However, different parts of the body produce various amounts, for example hands produce 80–160 g h^−1^, feet 50–150 g h^−1^, head and neck produce around 40–75 g h^−1^, and all remaining sites losing 15–60 g h^−1^.^[^ ^195^ ^]^ These studies predict that it will be advantageous to attach the sweat‐based energy autonomus sensory system in the above mentioned parts of the body.

Material engineering involving the development of various hydrogels that are customized for the sweat‐based devices, will be extremely helpful toward building a stable, practical, and energy‐autonomous sweat‐based system. For such practical applications it is highly necessary to design integrated system where electronics and related energy management components should be reusable. So, when designing the sweat monitoring sensors, energy generators and energy storage, the consideration of sustainable and eco‐friendly materials will be real advantage for disposable devices.^[^ ^73^, ^228^, ^229^, ^230^ ^]^

## 8 Conclusion

Energy autonomy is key to the next generation portable and wearable systems. In this regard, we have attempted to closely investigate the progress in the field of sweat‐based sensing and energy elements, with the goal of establishing self‐sustainable systems. Recent reports show that the power requirements of advanced sensing and communications can be in the order of several hundred µW. These requirements generally align with sweat‐based generators such as biofuel cells (with maximum power density 3.5 mW cm^−2^), and energy storage devices such as supercapacitors (with respective energy and power densities of 1.36 Wh kg^−1^ and 329.70 W kg^−1^ at 1.31 V), and sweat‐activated battery (with typically 67 Ah kg^−1^). These performance metrics are very encouraging and offer tremendous opportunities for the development of a self‐sustainable platform for wearables in applications, such as personalized health monitoring and sports. Meanwhile, the performance of epidermal energy harvesting and storage devices is yet to be enhanced to power high‐demand applications, such as prosthetics, light‐emitting displays, or interactive systems for augmented/virtual reality. There are several critical points that need to be considered for the development of an effective platform. These include the availability of sufficient sweat or sweat equivalent volumes and the generation of sufficient power coupled with good power management. While addressing these scientific and engineering challenges, we may be faced with opportunities. For example, pursuits to find ways to increase sweat volumes could also lead to the modeling of the skin or greater insight into sweat production, automated sweat extraction processes, and using the same to develop a virtual platform which could be subsequently used to simulate performance prior to the development of an actual system. This can provide a means to develop user‐centric solutions since sweat content varies with individuals and/or with physical activity. In addition, modeling in general can benefit those pursuing even broader types of on‐skin products, such as cosmetics, antiperspirants, garments, and medical adhesives. The sweat‐based approach could also help advance the prosthesis where sufficient sweat generated at the socket‐limb interface could be exploited to generate power and hence monitor the health conditions of the physically‐handicapped. The sweat is extracted from the people, sensed by the people, and shall be used toward storing and generating power for the people. We believe that the sweat‐based energy systems, leveraging their attractive features, such as high biocompatibility, availability, and rich biomarker information, is an inseparable integral in the next‐generation integrated systems that are truly safe, high‐efficiency, and self‐sustainable.

## Conflict of Interest

The authors declare no conflict of interest.
